# CD47 Aptamer-Decorated Fluorescent POSS Hybrid Nanoparticles as a Biohybrid Interface for Probing Prostate Cancer–Macrophage Interactions

**DOI:** 10.3390/biomimetics11080588

**Published:** 2026-08-18

**Authors:** Sumeyye Altunok, Gunes Kibar, Fatma Aylaz, Dide Su Demirel, Veli Cengiz Ozalp

**Affiliations:** 1 Medical School, Faculty of Medicine, Atılım University, 06830 Ankara, Türkiye; altunok.1@osu.edu (S.A.); gkibar@atu.edu.tr (G.K.); fatma.aylaz@atilim.edu.tr (F.A.); demirel.didesu@student.atilim.edu.tr (D.S.D.); 2 Department of Neurology, The Ohio State University Wexner Medical Center, Columbus, OH 43210, USA; 3 Micro Nano Particles (MNP) Research Group, Department of Materials Science and Engineering, Adana Alparslan Türkeş Science and Technology University, 01250 Adana, Türkiye; 4 Department of Biomedical Engineering, Faculty of Engineering and Natural Sciences, Istanbul Atlas University, 34408 Istanbul, Türkiye

**Keywords:** CD47–SIRPα axis, DNA aptamer, POSS-based nanoparticles, biohybrid interface, tumour–macrophage interactions, prostate cancer

## Abstract

Prostate cancer remains a major clinical challenge, partly due to tumour–immune interactions that contribute to immune evasion and therapeutic resistance. The CD47–SIRPα axis is a key macrophage-associated immune recognition pathway; and materials-oriented platforms that allow preliminary investigation of tumour–macrophage interaction patterns remain valuable. Here, we report an optically traceable biohybrid nanoplatform based on CD47 aptamer-functionalized fluorescent carboxyl-functional MMES-POSS hybrid nanoparticles. The nanoparticles were synthesized within 5 min using UV-induced free-radical emulsion polymerization in an ethanol–water system and exhibited spherical morphology, SEM-derived dry-state mean diameters below 100 nm, negative surface charge, and retained fluorescence due to RITC encapsulation within the crosslinked hybrid matrix; however, DLS measurements revealed pronounced aggregation and polydispersity in aqueous dispersion. SEM and EDX provided complementary evidence of nanoparticle morphology and elemental composition, whereas zeta potential and Nanodrop measurements provided evidence consistent with surface functionalization, and FTIR confirmed retention of the core POSS, carbonyl, and RITC-associated chemistry. In a PC-3/THP-1 macrophage co-culture model, Annexin V–FITC/PI flow cytometry showed a concentration-dependent redistribution of cell populations, with the most pronounced early apoptotic enrichment observed at 2 µg/mL. However, pairwise exploratory comparisons among concentrations did not reach statistical significance, and this is discussed as a limitation of the present exploratory dose–response characterization. These findings indicate preliminary interaction-associated response patterns rather than definitive receptor-specific targeting or therapeutic CD47–SIRPα blockade. Overall, this modular POSS-based biohybrid interface provides a materials-oriented platform for probing prostate cancer–macrophage interaction profiles and guiding future mechanistic validation studies.

## 1. Introduction

Prostate cancer is among the most frequently diagnosed malignancies in men and continues to represent a substantial clinical challenge, particularly because of its high clinical burden in older patient populations [1]. While radiotherapy, chemotherapy, and androgen deprivation therapy remain integral components of standard clinical management, their long-term effectiveness is frequently limited by therapeutic resistance, disease progression, and treatment-associated systemic toxicity [2,3,4]. These limitations highlight the need for innovative exploratory and materials-oriented approaches that can support the investigation of tumour–immune interactions within the tumour microenvironment [4,5,6]. From a biomimetic nanomaterials perspective, traceable biohybrid platforms are particularly valuable because they integrate engineered nanostructures with biologically derived recognition motifs to examine complex cellular communication events [7,8,9,10].

Within this immunoregulatory landscape, Cluster of Differentiation 47 (CD47) has emerged as a key immune checkpoint associated with tumour cell avoidance of macrophage-mediated clearance [11,12,13,14,15]. Engagement of CD47 with signal regulatory protein alpha (SIRPα) on macrophages delivers a potent “don’t eat me” signal, suppressing phagocytosis and facilitating immune escape [12,13,14]. The CD47–SIRPα axis therefore provides a biologically relevant model of immune self-recognition and macrophage avoidance, making it an informative biological axis for designing biohybrid systems that examine tumour–macrophage communication [5,13,15]. Although CD47-directed monoclonal antibodies have shown therapeutic promise, their clinical translation has been challenged by dose-limiting haematological toxicities and on-target effects on healthy cells [16,17,18]. These challenges have encouraged the exploration of alternative molecular recognition strategies that may offer greater tunability, modularity, and compatibility with nanoscale interface design [9,18,19,20].

Recent advances in CD47-oriented nanomedicine have demonstrated multiple strategies for engaging the CD47–SIRPα macrophage checkpoint axis [21,22]. For instance, anti-CD47-functionalized nanoparticles have been developed to combine checkpoint inhibition with calreticulin-mediated pro-phagocytic signalling, thereby reinforcing macrophage-associated antitumour responses. In parallel, immune-modulating nanocarriers have been investigated for the co-delivery of CD47 siRNA and immunostimulatory agents such as R848, aiming to attenuate CD47 expression, activate dendritic cells, and promote downstream antitumour immune responses [23]. Peptide- or CD47-conjugated nanoparticles and biomimetic membrane-derived nanovesicles further illustrate the breadth of nanoscale approaches designed to influence macrophage engulfment and CD47–SIRPα signalling [24,25].

Beyond CD47-specific systems, recent nanoparticle studies have also emphasized that biological performance is strongly influenced by nanoscale design parameters, including particle size, surface charge, colloidal behaviour, fluorescence stability, and ligand presentation [26,27]. Fluorescent and functional hybrid nanoplatforms have therefore been increasingly evaluated not only in terms of material formation, but also according to optical traceability, surface functionality, and biologically relevant response profiles [28]. Beyond fluorescence-based traceability alone, several recent multifunctional nanoplatforms have combined optical imaging with stimuli-responsive therapeutic delivery, including a pH/NIR dual-responsive implantable patch for combined photothermal and chemotherapeutic tumour treatment [29] and cyano-modulated Zn(II)-Schiff base complexes enabling lysosome-targeted fluorescence imaging together with pH-responsive camptothecin release [30], further illustrating the versatility of fluorescent nanomaterials as combined imaging and functional platforms. In addition, ligand-modified nanomaterials highlight the importance of comparing ligand density, surface chemistry, and cell-associated readouts when assessing targeted nanoscale biointerfaces [31].

Taken together, these studies demonstrate that nanoscale CD47-oriented and ligand-modified platforms can be engineered to influence macrophage-associated recognition, phagocytosis-related responses, and tumour-associated cellular interactions. Nevertheless, most reported CD47-oriented nanomedicine strategies have primarily focused on therapeutic blockade, cargo delivery, phagocytosis enhancement, or immune microenvironment remodelling. This leaves a conceptual and materials-oriented space for modular fluorescent nanoplatforms that combine molecular recognition motifs with compact, optically traceable, and chemically addressable biohybrid interfaces. Within this context, such platforms are particularly valuable for the preliminary investigation of tumour–macrophage interaction patterns, especially when the aim is to establish material design, traceability, and interaction-associated response profiles rather than to claim definitive receptor-specific blockade or therapeutic immune modulation.

Against this background, the combination of a CD47 aptamer-functionalized recognition interface with polyhedral oligomeric silsesquioxane (POSS)-derived hybrid nanostructures offers a rational design strategy for constructing compact and chemically addressable fluorescent biointerfaces. Aptamers provide programmable recognition motifs that can be selected against defined molecular targets and integrated into nanoscale systems without relying on antibody-based architectures [7,9,19,20,32]. POSS, in turn, provides a rigid Si–O–Si cage framework with tunable organic substituents, structural stability, and accessible functional groups for further chemical modification [33,34,35,36]. In the present design, carboxyl-functionalized fluorescent POSS was used as an optically traceable hybrid scaffold that could support biofunctionalization while preserving the advantages of a compact inorganic–organic architecture [10,33,34,37]. The ethanol-based rapid synthesis also provides an experimentally practical and biologically oriented fabrication route for preparing the fluorescent POSS platform.

Herein, we report the rapid ethanol-based synthesis, physicochemical characterization, and preliminary biological evaluation of a CD47 aptamer-functionalized fluorescent MMES-POSS nanoplatform designed for investigating tumour cell–macrophage interaction patterns in vitro. The study focuses on establishing the material design, fluorescence traceability, surface functionalization, and Annexin V/propidium iodide (PI)-based co-culture response profile of the platform rather than claiming definitive receptor-specific targeting or therapeutic CD47–SIRPα blockade. By positioning Apt-RITC@MMES-POSS as an exploratory biohybrid interface, this work provides a materials-oriented foundation for future studies aimed at directly validating molecularly guided tumour–macrophage interaction patterns.

## 2. Materials and Methods

### 2.1. Materials

The methacrylated POSS cage mixture (MA-0735) was obtained from Hybrid Plastics Inc. (Hattiesburg, MS, USA). The carboxyl-functional monomer MMES (454974), fluorescent dye rhodamine B isothiocyanate (RITC-283924), the UV photoinitiator 2-hydroxy-2-methylpropiophenone (Darocur 1173-405655), and the surfactant sodium dodecyl sulfate (SDS) were purchased from Sigma-Aldrich (St. Louis, MO, USA). Absolute ethanol was supplied by Merck A.G. (Darmstadt, Germany), and distilled deionized water was produced using a Millipore Direct-Q 3UV (Millipore Corp., Burlington, MA, USA) water purification system.

### 2.2. Synthesis of Carboxyl Functional Hybrid Fluorescent Nanoparticles

**Figure 1:**
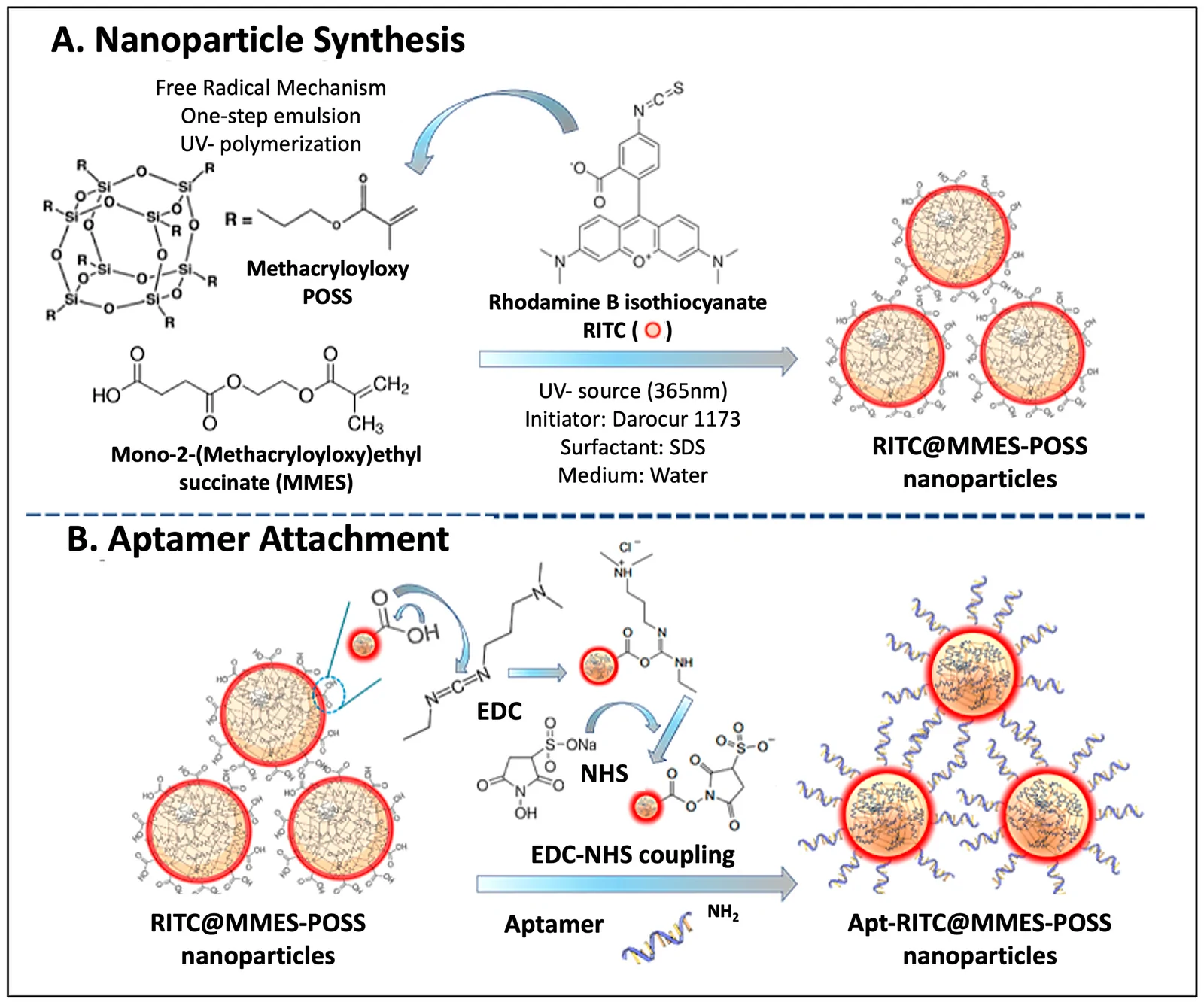
Schematic illustration of the synthesis and post-functionalization of fluorescent, carboxyl-functionalized POSS hybrid nanoparticles: (**A**) One-step UV-induced free-radical emulsion polymerization of methacryloxypropyl-POSS and the carboxyl-functional comonomer MMES in the presence of RITC, yielding fluorescent POSS-based hybrid nanoparticles with surface-exposed carboxyl groups. (**B**) Subsequent surface activation of carboxyl groups and covalent conjugation with amine-containing molecules via EDC/NHS chemistry, resulting in functionalized POSS nanoparticles decorated with aptamers as surface ligands. The red shell represents the functionalized polymeric POSS matrix still exhibiting fluorescent activity, while the inner network indicates the hybrid inorganic–organic structure.

Carboxyl-functionalized POSS nanoparticles were synthesized in an environmentally friendly manner via a one-step UV-induced free-radical emulsion polymerization (Figure 1A). Briefly, the inorganic–organic hybrid POSS monomer (25 mg) and the carboxyl-functional co-monomer mono-2-(methacryloyloxy)ethyl succinate (MMES, 25 mg) were mixed at a 1:1 weight ratio and dissolved in 1 mL of absolute ethanol containing RITC (0.5 mg/mL). The UV initiator Darocur 1173 (20 µL; Table 1) was added to the monomer mixture. This solution was emulsified in 10 mL of aqueous SDS solution (0.25%, *w*/*v*). The resulting emulsion was irradiated with a 10 W high-power UV-LED source (365 nm) for 5 min to initiate polymerization. After polymerization, the nanoparticles were collected by centrifugation at 15,000 rpm for 2 min, thoroughly washed several times with ethanol and deionized water, air-dried, and stored at 4 °C in the dark until further use.

### 2.3. CD47 Aptamer Conjugation on Carboxyl Functional Hybrid Fluorescent Nanoparticles

In this study, a CD47-specific DNA aptamer reported in the literature was employed [38]. This aptamer sequence was identified computationally via a stochastic tunnelling–basin hopping–discrete molecular dynamics simulation approach [38]; to our knowledge, independent experimental confirmation of its binding to CD47 (e.g., by surface plasmon resonance, ELONA, isothermal titration calorimetry, or a dedicated cell-binding assay) has not been reported in the original study or in any subsequent publication. Accordingly, the CD47-targeting capacity of this aptamer should be regarded as a computationally predicted, not experimentally validated, property, and all cell-association findings reported below are interpreted in that light. The aptamer was synthesized by Oligomer Biotechnology (Ankara, Türkiye) with an amino modification at the 5′ terminus and had the following sequence:

5′-ATA CCA GCT TAT TCA ATT CCC GAC CCA AGA TTG TAC CGC GGC CAA CGT CAC ACG GGC AAG TCC AGA CAT TTA AGA TAG TAA GTG CAA TCT-3′.

Covalent conjugation of the CD47 aptamer to the nanoparticles was performed using a carbodiimide coupling strategy, which is widely used for covalent attachment of amino-modified aptamers to carboxyl-functionalized nanoparticles to produce stable bioconjugates for targeted delivery applications in nanomedicine (Figure 1B) [7,37]. Briefly, 10 mg of nanoparticles were resuspended in reaction buffer, and surface carboxyl groups were activated by adding 5 µL of EDC (Bio-Rad, Hercules, CA, USA, ProteOn™ EDAC, Cat. No. 1762411) and 5 µL of sulfo-NHS (Bio-Rad, ProteOn™ Sulfo-NHS, Cat. No. 1762401). The mixture was gently stirred at room temperature to generate reactive NHS esters on the nanoparticle surface. Subsequently, the activated nanoparticles were incubated with the amino-modified CD47 aptamer, allowing the terminal amino groups of the aptamer to react with the NHS-activated carboxyl groups and form stable amide linkages.

The aptamer concentration was analysed using a Nanodrop 2000–3300 spectrophotometer (Thermo Fisher Scientific, Madison, WI, USA), and the amount of attached oligomer was calculated accordingly. Here, C(aptamer)_initial and C(aptamer)_residual denote the oligomer concentrations in the adsorption solution before and after nanoparticle incubation, respectively. m(nanoparticles) represents the mass (mg) of RITC@MMES-POSS nanoparticles used in the adsorption process, and V (mL) corresponds to the total volume of the adsorption solution. The calculated value reflects the amount of aptamer bound per unit mass of nanoparticles.

### 2.4. Characterization of Nanoparticles

The morphological features of the synthesized POSS nanoparticles were examined by scanning electron microscopy (SEM) using a Quanta 450 instrument (Akishima, Tokyo, Japan). For SEM analysis, the nanoparticle suspensions were first homogenized by vortex mixing, after which 2 µL of the dispersion was deposited onto double-sided conductive carbon tape affixed to a flat aluminium stub. The samples were allowed to air-dry for approximately 5 min and subsequently coated with a ~6 nm Au–Pt layer by sputtering to improve surface conductivity and minimize beam-induced charging and thermal damage during imaging. Elemental composition and surface chemical characteristics were further assessed by energy-dispersive X-ray spectroscopy (EDX) (FEI Company, Hillsboro, OR, USA) integrated into the SEM system.

The particle size and size distribution of the nanoparticles were determined from SEM micrographs using ImageJ software (version 1.54s, National Institutes of Health, Bethesda, MD, USA). For each formulation, a minimum of 200 individual nanoparticles were randomly selected and analysed to ensure statistical reliability. Particle diameters were measured using calibrated scale bars, and size distributions were expressed as mean diameter ± standard deviation.

Surface charge measurements were performed by determining the zeta potential of the nanoparticles in aqueous dispersion using a Zetasizer Nano ZS (Malvern Instruments, Malvern, UK). Measurements were conducted at 25 °C under standard operating conditions, and reported values represent the average of at least three independent measurements.

The fluorescence properties of the nanoparticles were characterized by fluorescence microscopy using a NEXCOPE NIB620FL system (Demir Sistem Lab., İstanbul, Türkiye) equipped with a 20× objective. To allow direct comparison among samples, all imaging parameters, including exposure time and fluorescence intensity, were kept constant throughout the measurements. Fluorescence images were collected using LED excitation sources optimized for the fluorophore; specifically, RITC-labelled nanoparticles were excited with a 525 nm LED source.

Fourier-transform infrared (FTIR) spectroscopy (Thermo Scientific Nicolet™, Madison, WI, USA) was utilized to investigate the chemical structures and functional groups of the synthesized fluorescent POSS nanoparticles. FTIR spectra were recorded in the range of 4000–500 cm^−1^ using powder-form samples.

### 2.5. In Vitro Co-Culture Treatment and Flow Cytometry Analysis

To evaluate cell-associated response patterns following exposure to CD47 aptamer-functionalized nanoparticles, monoculture and co-culture systems were established. PC-3 human prostate cancer cells (ATCC CRL-1435, Cell line kindly provided by Prof. Dr. Filiz Bakar Ates, Ankara University Faculty of Pharmacy; CD47 high-expression model) were maintained under standard culture conditions and seeded into 96-well plates at a density of 1 × 10^4^ cells per well. Cells were incubated at 37 °C in a humidified atmosphere containing 5% CO_2_ for 24 h to reach 70–80% confluence. Cells were then treated with the nanoparticle or antibody formulations described below and incubated for 4 h at 37 °C to allow surface binding, followed by three washes with phosphate-buffered saline to remove unbound nanoparticles or antibody. THP-1 human monocytic cells (ATCC TIB-202, Cell line kindly provided by Prof. Dr. Gunes Esendagli, Hacettepe University Faculty of Medicine) were then added onto the treated PC-3 cells at a 1:1 ratio, together with phorbol 12-myristate 13-acetate (PMA, 100 ng/mL) and ionomycin (0.5 µg/mL) to induce macrophage-like differentiation to induce macrophage-like differentiation, as previously described [39]. The combined co-culture was incubated for 48 h at 37 °C in a humidified atmosphere containing 5% CO_2_, allowing concurrent THP-1 differentiation, cell–cell interaction, and progression of nanoparticle/antibody-associated cellular responses. 

A set of experimental and control groups was included to compare aptamer-functionalized nanoparticles with aptamer-free nanoparticles and baseline cellular responses (Table 2); however, without a sequence-scrambled aptamer–nanoparticle control, these groups cannot distinguish sequence-dependent CD47 activity from nonspecific effects of surface functionalization. Negative controls included PC-3 cells cultured in medium alone (NC1) and PMA-differentiated THP-1 macrophages cultured in medium alone (NC2). Cell–cell interaction controls consisted of PC-3 and THP-1 co-cultures maintained in the absence of nanoparticles (NC3). Nanoparticle-related background signals were evaluated using PC-3/THP-1 co-cultures treated with non-aptamer-functionalized RITC@MMES-POSS nanoparticles (NC4). As a comparator for CD47-directed treatment, PC-3/THP-1 co-cultures treated with an anti-CD47 monoclonal antibody (Santa Cruz, B6H12) were included; this antibody comparator does not establish sequence-specific activity of the aptamer-functionalized nanoparticles. Experimental samples comprised PC-3/THP-1 co-cultures treated with Apt-RITC@MMES-POSS nanoparticles.

For the experimental group, aptamer-functionalized fluorescent nanoparticles (Apt-RITC@MMES-POSS) were prepared at defined concentrations using a two-fold serial dilution method in accordance with previously reported protocols [39,40,41]. Nanoparticle concentrations ranged from 128 µg/mL to 0.0625 µg/mL to enable evaluation of concentration-dependent cellular response.

For nanoparticle toxicity assessment, co-culture systems were exposed to the highest nanoparticle concentration (128 µg/mL) using non-aptamer-functionalized RITC@MMES-POSS nanoparticles. As a positive control confirming maximal CD47–SIRPα engagement, the co-culture system was treated with an anti-CD47 antibody at a dose of 1 µg per 2 × 10^4^ cells.

Following the 48 h co-culture period, cells were harvested and stained with Annexin V-Fluorescein isothiocyanate (FITC)/PI for 30 min in the dark (Elabscience, Wuhan, China, E-CK-A211-100A), following the manufacturer’s instructions. Flow cytometric analysis was performed using a CytoFLEX flow cytometer (Beckman Coulter, Brea, CA, USA), with excitation/emission wavelengths of 488/519 nm for FITC and 535/617 nm for PI. A minimum of 10,000 events per sample were acquired. Cell populations were classified as viable, early apoptotic, late apoptotic, or necrotic based on standard Annexin V/PI staining profiles.

### 2.6. Statistical Analysis

All experiments were performed in triplicate unless otherwise stated. Flow cytometry data were obtained from three technical replicates and are presented descriptively as mean values or mean ± standard deviation, where applicable. For the Annexin V–FITC/PI population distribution (Table 3), one-way ANOVA was additionally performed across all tested concentrations for each quadrant population to assess whether not all group means were equal (Supplementary Table S3). Selected pairwise Welch’s *t*-tests were conducted as exploratory comparisons; these tests were not corrected for multiplicity and are interpreted as hypothesis-generating rather than confirmatory. For all other physicochemical and biological measurements, results were interpreted descriptively to illustrate distributional changes between experimental conditions rather than to establish statistically significant differences. Flow cytometry analyses were performed using FlowJo software (version 11.2; BD Biosciences, San Jose, CA, USA).

## 3. Results and Discussion

### 3.1. Nanoparticle Synthesis and Characterization

**Figure 2:**
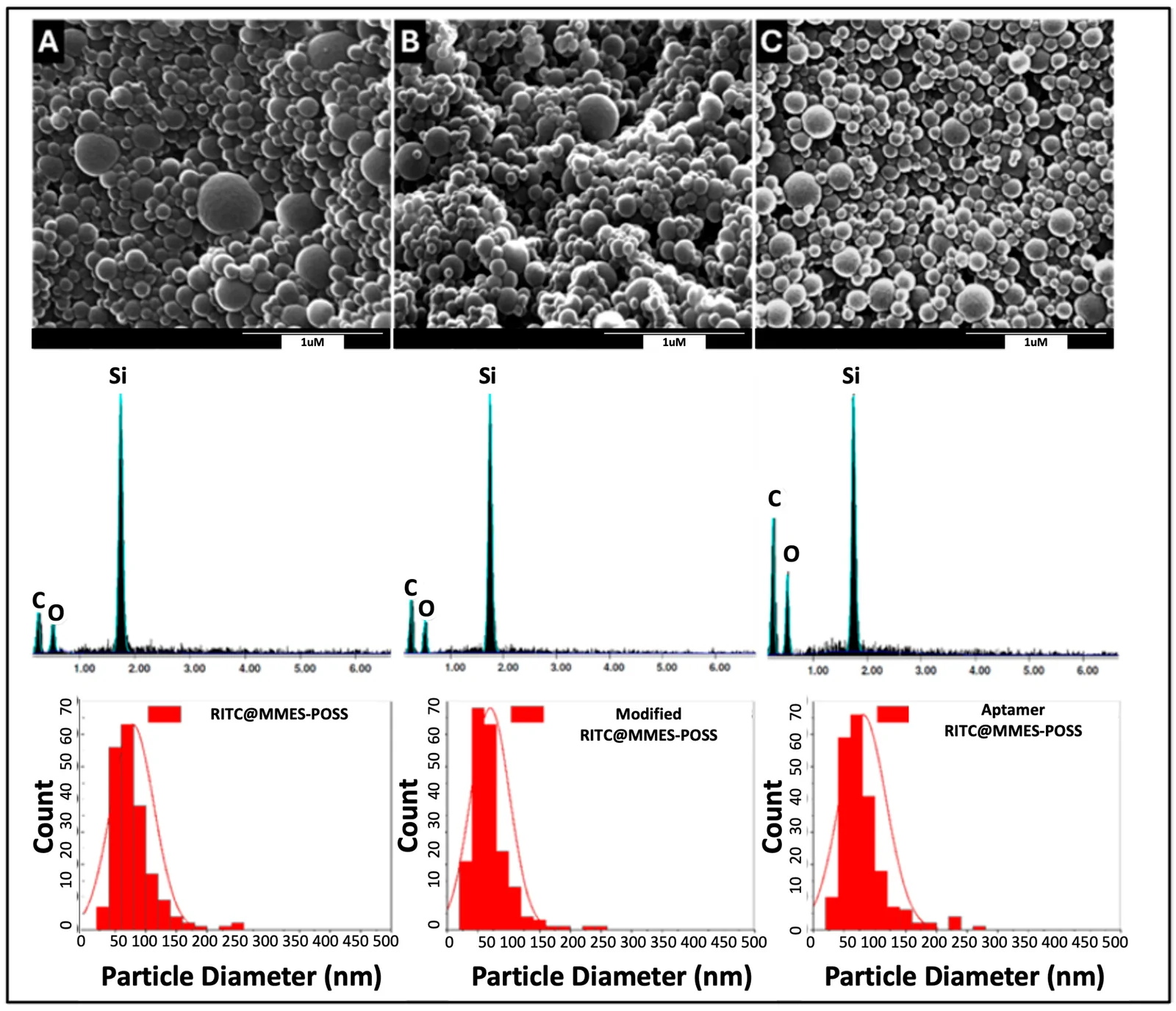
Morphological, elemental, and size distribution analysis of the synthesized nanoparticles. SEM images, EDX spectra, and particle size distribution histograms of (**A**) RITC@MMES-POSS nanoparticles, (**B**) surface-modified RITC@MMES-POSS nanoparticles, and (**C**) aptamer-functionalized RITC@MMES-POSS nanoparticles. SEM images show spherical particle morphology, EDX spectra indicate the elemental composition of the POSS-based hybrid structure, and histogram plots show the corresponding particle size distributions obtained from SEM image analysis.

Carboxyl-functionalized POSS nanoparticles were synthesized using an environmentally friendly green approach based on a one-step UV-induced free-radical emulsion polymerization. This strategy enables rapid fluorescence incorporation while avoiding harsh reaction conditions and excessive use of organic solvents, thereby offering an efficient, sustainable, and fast route for the preparation of fluorescent, functional POSS-based nanomaterials. The morphological characteristics of the nanoparticles were revealed with SEM analysis (Figure 2A–C in the top panel). All nanoparticle formulations—RITC@MMES-POSS, surface-modified RITC@MMES-POSS, and aptamer-functionalized RITC@MMES-POSS—exhibited predominantly spherical dry-state morphology, together with visible clustering and size heterogeneity. SEM analysis was employed to evaluate dry-state particle morphology and approximate size distributions, and is not suitable for resolving thin organic surface layers due to the presence of conductive Au–Pt coating and the low electron density of organic materials.

During UV-induced free-radical emulsion polymerization, nanoparticle formation occurs within surfactant-stabilized micellar domains, where POSS and MMES monomers undergo rapid radical-initiated crosslinking. The spherical morphology arises from the minimization of interfacial energy within the emulsion droplets, leading to the formation of thermodynamically favourable structures. Furthermore, the enrichment of carboxylic acid groups on the nanoparticle surface can be attributed to the preferential orientation of MMES monomers toward the aqueous interface during polymerization, resulting in surface-exposed functional groups suitable for subsequent conjugation.

RITC does not directly participate in the polymerization reaction but is physically encapsulated within the forming polymer matrix during nanoparticle nucleation and growth. The incorporation of RITC is driven primarily by hydrophobic interactions with the organic domains of the MMES-POSS hybrid structure, allowing stable entrapment within the crosslinked network. This encapsulation mechanism contributes to the retention of fluorescence while preventing dye leakage during subsequent surface modification steps.

The preservation of particle morphology following surface modification and aptamer conjugation indicates that the post-synthetic functionalization steps did not disrupt the structural integrity of the POSS-based framework [37]. The UV-induced free-radical emulsion polymerization strategy enabled rapid particle formation under mild conditions; however, the broad SEM-derived size distributions and DLS evidence of aggregation do not support claims of controlled nucleation or growth [42]. The observed preservation of spherical morphology following functionalization should therefore be interpreted as dry-state morphological retention rather than evidence of particle-size control or reproducible dispersion behaviour. These colloidal limitations may influence nanoparticle–cell association, dispersion behaviour, and nano–bio interfacial interactions within complex biological environments [43].

The elemental composition of the synthesized nanoparticles before and after surface modification was analysed by EDX (Figure 2A–C in the middle panel), with quantitative results summarized in Supplementary Table S1. The EDX spectra of pristine RITC@MMES-POSS nanoparticles confirmed the presence of silicon (Si), oxygen (O), and carbon (C), consistent with the hybrid inorganic–organic architecture of the POSS-based system. The relatively high silicon content reflects the integrity of the silsesquioxane cage structure, while the carbon and oxygen signals originate from the methacrylate backbone [35], MMES units, and incorporated RITC fluorophore.

Following surface modification and aptamer conjugation, a progressive increase in carbon and oxygen content was observed, accompanied by a relative decrease in silicon contribution. No distinct nitrogen (N) or phosphorus (P) signals were detected, which can be attributed to the limited sensitivity of EDX for low-abundance surface-bound biomolecules and the low atomic fraction of these elements relative to the dominant Si–O framework.

Given that EDX primarily probes the bulk composition rather than surface-confined species, the absence of clear N and P signals does not exclude successful aptamer conjugation, but it also means that EDX cannot provide direct, diagnostic confirmation of a thin aptamer overlayer. Instead, the observed compositional shifts, particularly the relative increase in carbon and oxygen signals, are consistent with, but not definitive proof of, the introduction of organic surface components.

In particular, the Apt-RITC@MMES-POSS formulation exhibited the highest carbon (59.31 wt%) and oxygen (24.16 wt%) levels, indicative of successful surface enrichment with organic biomolecular components. Given the relatively low aptamer surface loading (approximately 40 ng aptamer per mg nanoparticle, as determined by Nanodrop analysis) and the absence of a distinct nitrogen or phosphorus signal, which would be the most diagnostic markers of nucleic acid attachment, this compositional shift cannot be specifically attributed to the introduction of the DNA aptamer and is more likely to reflect the preceding carboxyl-functionalization and surface modification steps. The systematic reduction in the Si signal after surface modification further supports the formation of an organic surface layer rather than degradation of the inorganic core. Collectively, the EDX data provide supportive compositional trends consistent with overall surface modification, rather than aptamer-specific or definitive evidence, complementing the morphological and spectroscopic characterization results. Surface-sensitive techniques such as X-ray photoelectron spectroscopy (XPS), which probes only the outermost ~5–10 nm of the surface and would enable direct detection of N 1s and P 2p signals diagnostic of nucleic acids, were not performed in this study but would provide more direct confirmation of aptamer surface attachment in future work. These findings suggest that the functionalization strategy preserves the structural integrity of the POSS framework while introducing surface chemical features compatible with subsequent biofunctionalization and cell-associated interaction studies.

The particle size distributions (Figure 2 in the bottom panels) revealed average diameters of 69.70 ± 31.74 nm, 78.45 ± 33.97 nm, and 80.25 ± 37.83 nm for RITC@MMES-POSS, surface-modified RITC@MMES-POSS, and Apt-RITC@MMES-POSS nanoparticles, respectively. These correspond to relative standard deviations of approximately 46%, 43%, and 47%, respectively. All formulations therefore exhibited broad size distributions rather than tightly controlled particle-size populations. This degree of polydispersity, together with the subpopulation of larger particles/aggregates visible in the SEM images (Figure 2A–C), represents a limitation for nanoparticle–cell interaction studies. While a slight increase in mean particle size is observed, these differences fall within experimental variability, particularly considering the relatively large standard deviations and the presence of the ~6 nm Au–Pt coating applied for SEM imaging; therefore, such variations cannot be reliably attributed to the formation of a thin organic layer or surface functionalization. Although SEM images (Figure 2A–C, top panels) show apparent particle clustering and size heterogeneity, drying and deposition during sample preparation may contribute to the observed clustering; however, the DLS results demonstrate that substantial aggregation also occurs in aqueous dispersion. Accordingly, the clustering observed by SEM cannot be attributed exclusively to dry-state preparation artefacts. Dynamic light scattering (DLS) measurements of RITC@MMES-POSS nanoparticles dispersed in water (*n* = 3) yielded Z-average hydrodynamic diameters of 4161, 1994, and 1434 nm, with correspondingly high polydispersity indices (PdI = 1.000, 1.000, and 0.891, respectively; flagged by the instrument as low-confidence cumulant fits). Although intensity-weighted Z-average values are particularly sensitive to larger aggregates and dust particles, the magnitude and replicate-to-replicate variability of the Z-average diameters and PdI values demonstrate severe aggregation and polydispersity in aqueous dispersion. The number-weighted distributions showed peaks at 44.1, 177.0, and 244.1 nm across the three replicates; however, the substantial variation among these values indicates poor between-replicate consistency and should not be interpreted as confirmation of a reproducible predominant particle size. These results, together with the SEM data, support the polydispersity already noted above as a limitation of the present platform. DLS summary parameters and the dominant number-weighted peak for each replicate are provided in Supplementary Table S2. Furthermore, at the concentrations used for in vitro cell culture experiments, the nanoparticles were dispersed in culture medium and did not exhibit macroscopically visible aggregation; however, this represents only a qualitative, low-sensitivity assessment and does not rule out the aggregation/polydispersity captured by DLS in aqueous dispersion (see above). Colloidal behaviour in complete culture medium may also differ from that in water due to protein corona formation, which can either stabilize or destabilize nanoparticle dispersions. The observed aggregation, polydispersity, and between-replicate variability therefore limit the reproducibility of nanoparticle–cell association and must be considered when interpreting the biological response data.

Although the SEM-derived dry-state mean diameters were below 100 nm, the broad SEM distributions and DLS evidence of aggregation preclude interpretation of the platform as size-controlled or colloidally uniform. Because particle size and size distribution can influence nano–bio interfacial interactions [44], the observed heterogeneity should be considered a limitation when interpreting the cell-associated response data rather than evidence of reproducible nanoparticle–cell association.

Surface functionalization was evaluated based on changes in zeta potential and quantitative Nanodrop analysis. RITC@MMES-POSS nanoparticles exhibited a negative surface charge of −30.11 mV, attributable to surface-exposed carboxyl groups. Following EDC–NHS–mediated aptamer conjugation, the zeta potential shifted to a slightly more negative value (−32.36 mV), which may be associated with the introduction of negatively charged phosphate groups from the DNA aptamer backbone. However, the magnitude of this change is relatively small and may fall within the typical variability of zeta potential measurements. Therefore, the shift should be considered supportive but not definitive evidence of surface modification.

Surface-associated aptamer loading was estimated using Nanodrop depletion analysis alongside zeta-potential measurements. The Nanodrop-based depletion calculation yielded an estimated aptamer loading of approximately 40 ng per mg of nanoparticles; however, this indirect estimate does not confirm covalent conjugation and cannot exclude losses caused by nonspecific adsorption or sample handling. The zeta-potential values obtained from three replicate measurements provide complementary but non-definitive evidence consistent with surface modification.

**Figure 3:**
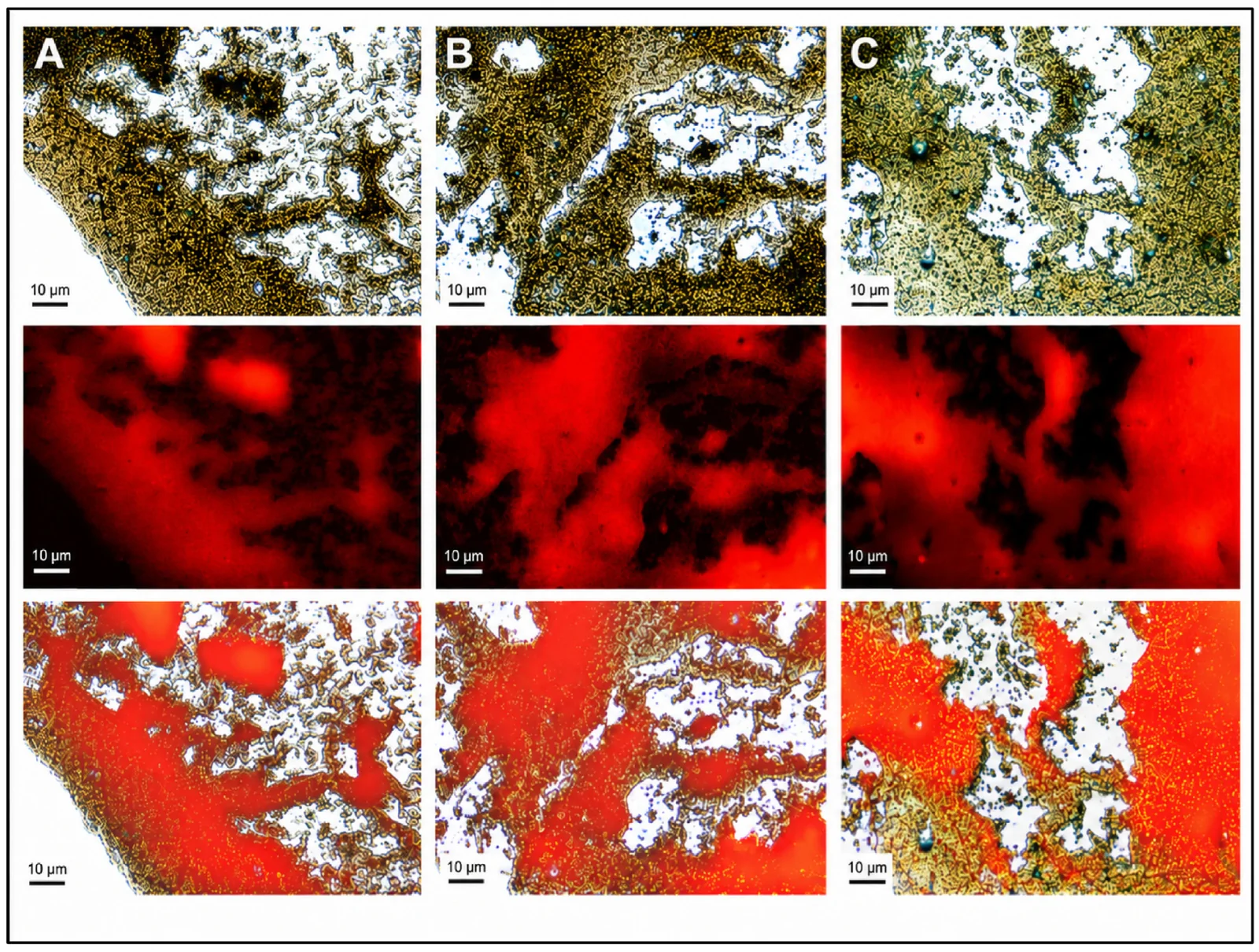
Fluorescence microscopy images of the synthesized nanoparticles: (**A**) RITC@MMES-POSS nanoparticles, (**B**) surface-modified RITC@MMES-POSS nanoparticles, and (**C**) aptamer-functionalized RITC@MMES-POSS nanoparticles. All images were acquired at 20× magnification; scale bar: 1 µm. Nanoparticles were deposited onto glass slides by drop-casting and imaged after air drying. The images represent fluorescence emission at the nanoparticle population level and do not reflect cellular uptake or single-particle resolution.

The fluorescence microscopy images of the RITC@MMES-POSS nanoparticles before and after surface modification and aptamer conjugation provide visual evidence supporting fluorophore incorporation and retention at the nanoparticle population level (Figure 3A–C). All nanoparticle formulations exhibited strong red fluorescence at the population level, supporting the successful incorporation of RITC within the POSS-based hybrid matrix during the UV-induced polymerization step [36]. Notably, fluorescence intensity and distribution were preserved following EDC–NHS–mediated surface modification and CD47 aptamer conjugation, indicating that post-synthetic functionalization did not compromise the photophysical integrity of the embedded fluorophore. Fluorescence microscopy provides qualitative, population-level information and was not used for quantitative or single-particle analysis.

The retention of RITC-derived fluorescence can be attributed to the stable encapsulation of RITC within the crosslinked MMES-POSS hybrid matrix. The rigid Si–O–Si framework restricts molecular motion and reduces non-radiative decay pathways, thereby minimizing fluorescence quenching. Additionally, the polymer network formed during UV-induced polymerization provides a protective microenvironment that prevents dye leakage and photobleaching.

The fluorescence observed in Figure 3 confirms bulk RITC retention within the nanoparticle population after synthesis and surface functionalization; because the images were obtained from cell-free, drop-cast samples, they do not provide evidence of nanoparticle–cell association.

Given the resolution limits of fluorescence microscopy, individual nanoparticles cannot be resolved in Figure 3, and no single-particle quantitative analysis is provided. Therefore, the observed fluorescence should be interpreted as a bulk or ensemble-level signal rather than evidence of single-particle homogeneity.

Accordingly, the fluorescence images indicate stable fluorophore incorporation and retention at the nanoparticle population level, rather than uniformity at the single-particle scale.

Consequently, Figure 3 should be interpreted solely as evidence of bulk RITC retention at the nanoparticle-population level after synthesis and surface functionalization. Evidence regarding nanoparticle–cell interaction is derived independently from the co-culture flow cytometry data presented in Section 3.2.

**Figure 4:**
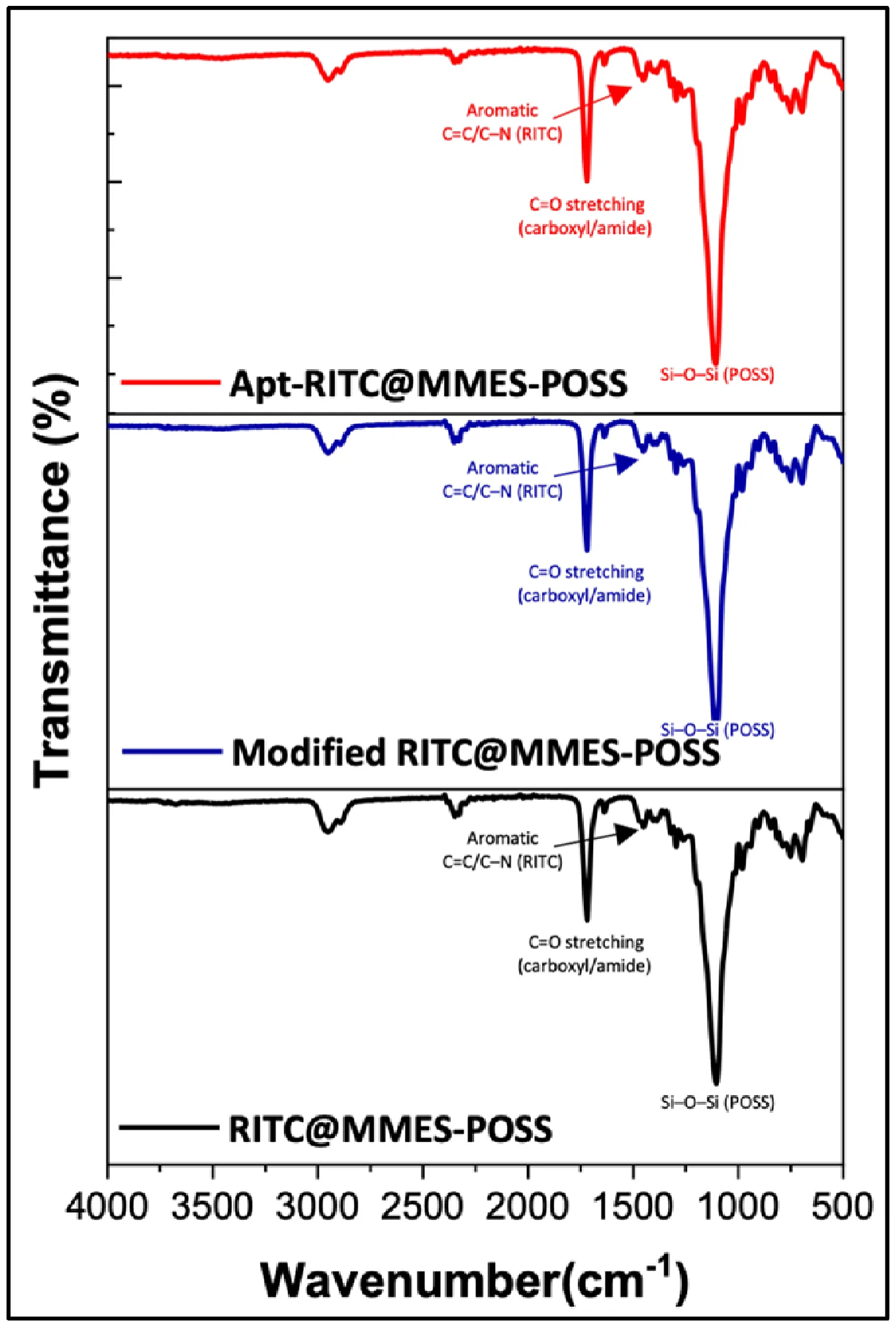
FTIR spectra of fluorescent hybrid nanoparticles. The spectra confirm retention of the core POSS, carbonyl, and RITC-associated chemistry after post-synthetic processing; they do not provide evidence of surface functionalization or aptamer conjugation.

The chemical structure and functional groups of the fluorescent nanoparticles, before and after surface modification and CD47 aptamer conjugation, were analysed by FTIR spectroscopy to assess retention of the underlying POSS-based core chemistry following post-synthetic processing (Figure 4). All nanoparticle formulations exhibited characteristic absorption bands associated with the silsesquioxane framework, including the strong Si–O–Si stretching vibration in the range of ~1080–1120 cm^−1^, confirming the structural integrity of the inorganic POSS cage following UV-induced polymerization and subsequent surface reactions [35]. The presence of C–H stretching vibrations at ~2850–2950 cm^−1^ further reflects the organic methacrylate backbone and MMES-derived polymer network [43].

For RITC@MMES-POSS nanoparticles, characteristic bands corresponding to ester carbonyl (C=O) stretching at ~1720–1735 cm^−1^ were observed, arising from the methacrylate and succinate functionalities, together with additional aromatic-associated vibrations in the ~1500–1600 cm^−1^ region attributable to the RITC fluorophore [44]. These features are consistent with fluorophore incorporation within the hybrid matrix. Following surface modification and EDC–NHS activation, no clear, unambiguous spectral differences attributable specifically to surface modification could be reliably distinguished from the baseline spectrum given the overall visual similarity of the three stacked spectra. Importantly, no new peaks indicative of polymer backbone degradation or POSS cage disruption were observed, underscoring the chemical robustness of the nanoplatform during post-synthetic processing. FTIR in this study is therefore presented only as confirming retention of the core POSS cage, carbonyl, and RITC-associated bands and the absence of polymer degradation after functionalization; surface modification and aptamer conjugation are instead evidenced by the Nanodrop and zeta potential measurements (Section 3.1), not by FTIR.

Upon CD47 aptamer conjugation, the Apt-RITC@MMES-POSS spectrum exhibited minor spectral variations, including slight intensity changes in the ~1600–1700 cm^−1^ and ~1000–1150 cm^−1^ regions. However, given the low aptamer loading achieved on the nanoparticle surface (approximately 40 ng aptamer per mg nanoparticle, as estimated by Nanodrop depletion analysis), these minor variations fall below the loading level typically required for FTIR to resolve a distinct, unambiguous nucleic acid-specific signature amid spectral overlap with the polymer matrix and POSS-related bands. Therefore, the FTIR results do not provide evidence of aptamer conjugation or surface functionalization and confirm only retention of the core POSS, carbonyl, and RITC-associated chemistry. Independent evidence consistent with surface functionalization is provided by the zeta potential and Nanodrop measurements.

Collectively, the FTIR data confirm retention of the hybrid POSS framework, carbonyl functionality, and RITC-associated bands after post-synthetic processing, but do not support conclusions regarding surface functionalization or aptamer conjugation.

Independent zeta potential and Nanodrop measurements provide evidence consistent with surface functionalization, whereas the FTIR analysis is restricted to confirming retention of the core chemical structure.

### 3.2. Annexin V/PI-Based Evaluation of Tumour–Macrophage Co-Culture Response Patterns Associated with Aptamer-Functionalized Nanoparticles

To evaluate whether CD47 aptamer-functionalized nanoparticles are associated with changes in tumour–macrophage co-culture response patterns, Annexin V–FITC/PI flow cytometry analysis was performed in a PC-3/THP-1 co-culture model representing a macrophage-containing tumour–immune interaction environment. Macrophage–tumour cell contact plays a central role in regulating apoptotic progression and immune-mediated clearance; therefore, the analysis focused on treatment-associated redistribution of cellular populations rather than absolute quantification of cell death. Cells were categorized into viable, early apoptotic, late apoptotic, and necrotic fractions based on phosphatidylserine exposure and membrane integrity, and representative distribution patterns across the technical replicates are summarized descriptively in Table 3. Under untreated co-culture conditions, macrophage–tumour interaction alone altered the equilibrium between viable and apoptotic populations (Supplementary Figure S1), supporting the use of this model for assessing interaction-associated cellular response patterns.

As shown in Figure S1, a measurable level of early apoptotic cells is also observed in monoculture conditions, particularly in THP-1 cells. This can be attributed to the inherent sensitivity of suspension-derived monocyte/macrophage-like cells to environmental stress and handling conditions, as well as the high sensitivity of Annexin V staining in detecting early membrane changes.

In the co-culture system, the increased apoptotic fraction likely reflects basal macrophage–tumour cell interactions, where partial immune recognition may occur even in the absence of aptamer-functionalized nanoparticle treatment. Such baseline activity is expected in innate immune interaction models and does not represent treatment-induced cytotoxicity.

Treatment with aptamer-free RITC@MMES-POSS nanoparticles did not produce a notable reduction in overall viability compared with untreated co-culture controls (Supplementary Figure S2). These observations are based on Annexin V/PI population analysis and should be interpreted as changes in apoptotic distribution rather than a comprehensive cytotoxicity assessment. We emphasize that a dedicated cytocompatibility assay (e.g., CCK-8, MTT, or Live/Dead staining) was not performed on PC-3 or THP-1 cells in isolation to establish baseline nanoparticle toxicity independent of the co-culture and Annexin V/PI readout, and this is acknowledged as a limitation of the present study. For context, we note that our group has independently reported favourable in vitro cytocompatibility (MTT viability >80% across 0–200 µg mL^−1^, confirmed by AO/PI staining, 24 h exposure) for a closely related RITC-functionalized POSS hybrid nanoparticle platform evaluated in HepG2 cells; while this does not substitute for a dedicated assay on PC-3/THP-1 in the present system, it provides supporting precedent for the biocompatibility of this nanoparticle chemistry.

Nonetheless, this interpretation is consistent with recent evidence showing favourable in vitro cytocompatibility and limited nonspecific toxicity for comparable hybrid nanoparticle systems. Instead, redistribution toward late apoptotic populations was consistent with basal co-culture dynamics rather than direct nanoparticle-induced membrane damage, indicating that the hybrid POSS nanoplatform did not exhibit dominant nonspecific cytotoxic behaviour under the tested conditions.

**Figure 5:**
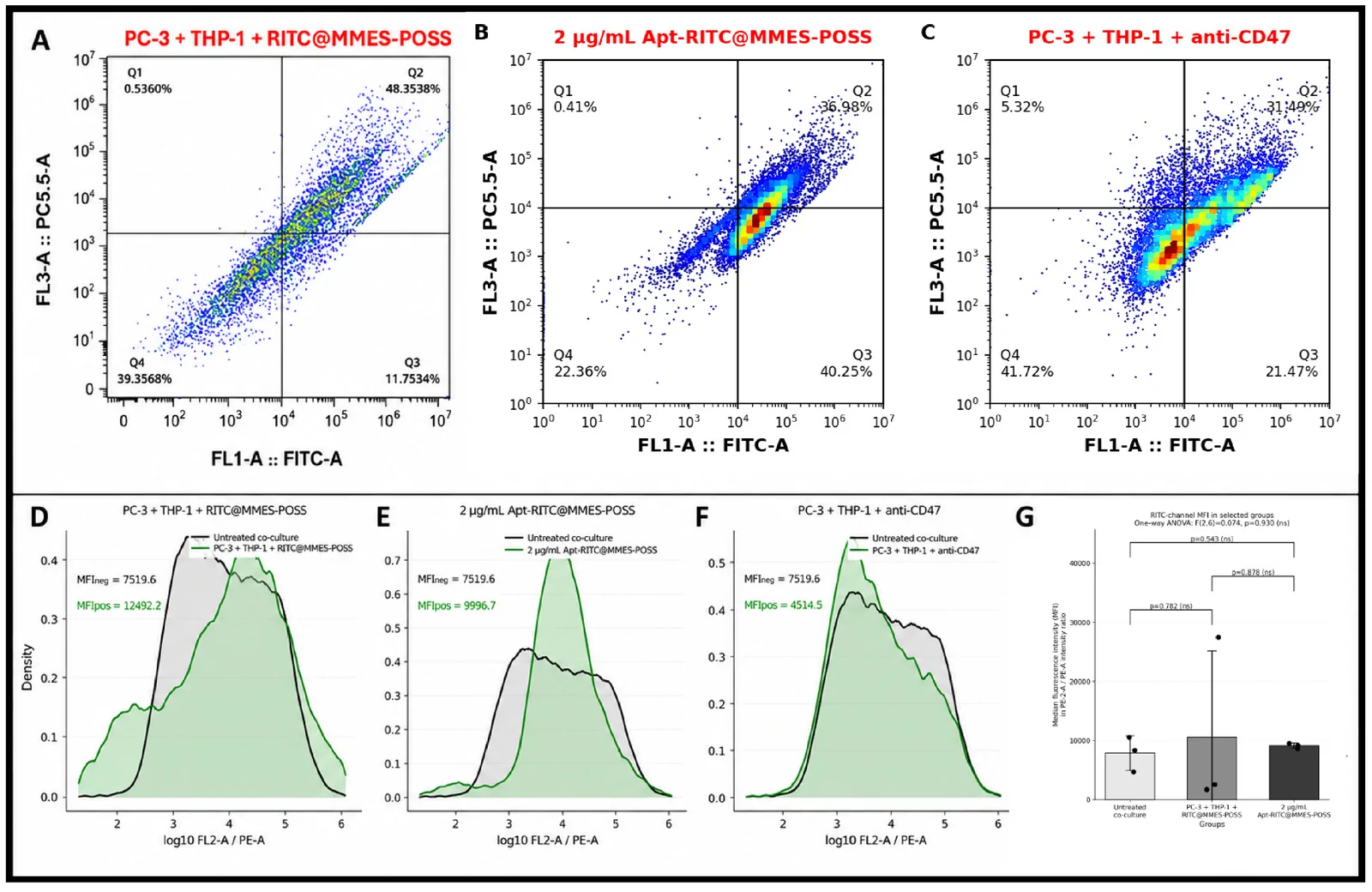
Representative flow cytometry and RITC-channel fluorescence analysis of PC-3/THP-1 co-cultures. (**A**–**C**) Annexin V–FITC/PI flow cytometry plots of co-cultures treated with RITC@MMES-POSS nanoparticles, 2 µg/mL Apt-RITC@MMES-POSS, and anti-CD47 antibody, respectively. Quadrants indicate viable cells (Q4), early apoptotic cells (Q3), late apoptotic/secondary necrotic cells (Q2), and necrotic cells (Q1). (**D**–**F**) Representative RITC-channel fluorescence histogram overlays for the corresponding groups, with untreated co-culture shown in black and treated samples shown in green. (**G**) RITC-channel MFI analysis calculated from the FL2-A/PE-A channel and presented as mean ± SD from technical replicates. MFI data were used as supportive evidence of fluorescence-associated cellular changes and were not interpreted as definitive proof of receptor-specific CD47 binding. Panels (**A**–**F**) show one representative measurement for each illustrated group. Quantitative summaries for the treated groups are based on three technical replicates and are reported as mean ± SD in Table 3; the untreated co-culture value is based on a single measurement.

Following exposure to Apt-RITC@MMES-POSS nanoparticles, a concentration-dependent but non-linear redistribution of cell populations was observed. At low nanoparticle concentrations (≤0.25 µg/mL), most cells remained within the viable fraction. Increasing the concentration into the intermediate range (0.5–2 µg/mL) produced a pronounced shift toward apoptotic populations, with ~36.6% early apoptotic enrichment most evident at 2 µg/mL (Figure 5 and Table 3). Although this value represents the numerically highest early apoptotic enrichment observed, it did not differ significantly from the immediately adjacent concentrations (1 µg/mL, *p* = 0.32; 4 µg/mL, *p* = 0.075; Welch’s *t*-test, Supplementary Table S3). This enrichment of Annexin V^+^/PI^−^ cells occurred without proportional accumulation of necrotic populations, suggesting an interaction-associated cellular response rather than acute membrane disruption. Further increases in nanoparticle concentration (4–128 µg/mL) did not enhance the apoptotic response and instead resulted in a plateau-like behaviour (Supplementary Figure S3), consistent with a non-linear interaction-associated response rather than an increasing cytotoxic burden [45]. One-way ANOVA confirmed a statistically significant overall effect of concentration on the distribution of all four quadrant populations (Q1: F(11,24) = 16.0, *p* < 0.0001; Q2: F(11,24) = 15.6, *p* < 0.0001; Q3: F(11,24) = 10.6, *p* < 0.0001; Q4: F(11,24) = 18.7, *p* < 0.0001; Supplementary Table S3). This result indicates that not all group means are equal but does not, by itself, establish that the observed non-monotonic pattern reflects a genuine biological effect rather than technical variability, plate-to-plate effects, or flow cytometry gating artefacts, none of which can be excluded with the present experimental design. For example, the transient increase in the viable population at 0.25 µg/mL differed from both the immediately lower (0.125 µg/mL, *p* = 0.0056) and higher (0.5 µg/mL, *p* = 0.0042) concentrations, while these two flanking concentrations did not differ from one another (*p* = 0.91). This response profile indicates that the system does not follow a conventional dose-dependent cytotoxic pattern. Instead, the observed peak at intermediate concentrations may reflect concentration-dependent changes in nanoparticle availability, surface interaction, steric effects, aggregation-related behaviour, or co-culture-associated cellular dynamics. However, receptor saturation cannot be concluded without direct binding, receptor competition, or CD47-silencing experiments. These and all other pairwise Welch’s *t*-tests reported in this section are exploratory and were not corrected for multiple comparisons; given the number of comparisons performed and the small per-group sample size (*n* = 3), they should be interpreted as hypothesis-generating rather than confirmatory.

In representative Annexin V–FITC/PI flow cytometry plots, the RITC@MMES-POSS nanoparticle control group was mainly distributed between viable and late apoptotic populations, whereas treatment with Apt-RITC@MMES-POSS at 2 µg/mL produced a pronounced enrichment of early apoptotic cells accompanied by a reduction in the viable fraction (Figure 5A,B). The anti-CD47-treated positive control also showed redistribution of Annexin V/PI populations, but the early apoptotic fraction was lower than that observed for the 2 µg/mL Apt-RITC@MMES-POSS group (Figure 5C). Because no sequence-scrambled aptamer–nanoparticle control or direct binding assay was included, this numerical difference cannot be attributed to ligand architecture, multivalent surface presentation, nanoscale interaction geometry, or sequence-dependent CD47 activity. To evaluate whether this difference reflects a genuine biological effect rather than assay variability, we compared the early apoptotic (Q3) population across the untreated co-culture (11.8%), the aptamer-free RITC@MMES-POSS nanoparticle control (17.2 ± 5.1%; Supplementary Figure S2), and the 2 µg/mL Apt-RITC@MMES-POSS group (36.6 ± 12.7%). This numerical ordering is equally compatible with nonspecific effects of surface functionalization or sampling variability, and pairwise comparisons among these groups did not reach statistical significance (Welch’s *t*-test: aptamer-free NP vs. 2 µg/mL Apt-NP, *p* = 0.10), reflecting the limited statistical power available with *n* = 3 replicates per group and the relatively high inter-replicate variability at 2 µg/mL. Accordingly, the present design cannot distinguish sequence-dependent CD47 activity from nonspecific nanoparticle-induced membrane perturbation, effects of surface functionalization, sampling variability, or assay-related factors such as differences in incubation time and readout between the antibody-based and nanoparticle-based treatments. A sequence-scrambled aptamer-nanoparticle control, which would more directly isolate sequence-specific effects from generic surface effects, was not included in the present study and represents an important addition for future validation. These findings support a treatment-associated shift in co-culture population distribution, with the strongest early apoptotic response observed at the intermediate aptamer-functionalized nanoparticle concentration.

RITC-channel fluorescence histogram analysis further showed detectable fluorescence-associated signals in nanoparticle-treated co-cultures (Figure 5D–F). The RITC@MMES-POSS nanoparticle control exhibited measurable cell-associated fluorescence, and the 2 µg/mL Apt-RITC@MMES-POSS group also showed a broadly comparable RITC-channel fluorescence profile relative to the untreated co-culture control. To provide a broader overview of fluorescence-associated changes across the full concentration range, overlay MFI histograms and a summary bar graph were additionally presented for all Apt-RITC@MMES-POSS concentrations and control groups (Supplementary Figure S4). This supplementary analysis showed detectable but non-linear RITC-channel fluorescence patterns across the dose series, with variability among selected treatment groups, particularly in the RITC@MMES-POSS nanoparticle control. Quantitative MFI analysis summarized these fluorescence-associated changes across the selected treatment groups; however, the RITC@MMES-POSS control displayed high variability among technical replicates (Figure 5G and Supplementary Figure S4). A one-way ANOVA comparing replicate-level median RITC-channel MFI values across the untreated co-culture, RITC@MMES-POSS (aptamer-free), and 2 µg/mL Apt-RITC@MMES-POSS groups did not reveal a statistically significant difference (F(2,6) = 0.074, *p* = 0.93), nor did pairwise comparisons between any two groups (Welch’s *t*-test, all *p* > 0.5). Therefore, the MFI data were interpreted as supportive evidence of fluorescence-associated nanoparticle–cell interaction rather than as proof of enhanced aptamer-mediated binding or receptor-specific CD47 targeting. Notably, the RITC-channel MFI and Annexin V/PI apoptotic responses did not necessarily converge on the same concentration. This divergence between fluorescence-based and functional readouts indicates that no single concentration can be identified as a definitive optimal dose within the tested range, and underscores the preliminary, exploratory nature of the present dose–response characterization.

The predominance of early apoptotic populations together with the absence of extensive necrotic accumulation is consistent with an interaction-associated cellular response rather than acute cytotoxic damage. In macrophage-containing systems, cell–cell contact can promote progression toward apoptotic states [46]. Because a sequence-scrambled aptamer–nanoparticle control and direct binding measurements were not included, the observed redistribution cannot be assigned to CD47-specific or sequence-dependent activity and may also reflect nonspecific effects of surface functionalization or sampling variability. Ligand multivalency, receptor clustering, and downstream signalling were not directly assessed and therefore cannot be invoked as mechanistic explanations in the present study.

The present findings are derived from flow cytometry-based functional analysis and therefore provide indirect evidence of nanoparticle–cell interaction dynamics rather than direct visualization of nanoparticle localization or receptor-specific binding. While imaging techniques such as confocal microscopy could further support these observations, the current results reflect biologically relevant cellular responses within the co-culture system.

Collectively, the Annexin V/PI and RITC-channel fluorescence data suggest that CD47 aptamer-functionalized POSS hybrid nanoparticles are associated with concentration-dependent changes in tumour–macrophage co-culture response patterns based on indirect functional readouts. The observed redistribution is consistent with interaction-associated cellular responses rather than direct cytotoxic or lytic mechanisms. However, in the absence of receptor competition, CD47 knockdown or knockout, uptake imaging, phagocytosis assays, cytokine profiling, or antigen-presentation analysis, these findings should be interpreted as preliminary evidence of nanoparticle–cell interaction dynamics rather than definitive proof of receptor-specific targeting or immune checkpoint modulation.

## 4. Conclusions

In this study, we present a rapidly fabricated and environmentally sustainable carboxyl-functional fluorescent nanoplatform based on MMES–POSS hybrid nanoparticles with SEM-derived dry-state mean diameters below 100 nm and spherical morphology; however, DLS measurements demonstrated substantial aggregation and polydispersity in aqueous dispersion. The ultrafast green synthesis strategy yields optically stable nanoparticles with characterizable morphology, surface chemistry, and surface charge, but not a controlled or monodisperse size distribution, as verified through complementary physicochemical characterization. Fluorescence observed at the nanoparticle population level indicates stable dye incorporation, ensuring reproducible optical performance following biofunctionalization and supporting qualitative optical tracking in biological systems.

Surface functionalization with a CD47-specific DNA aptamer produced an optically traceable biohybrid interface rather than a definitively validated receptor-specific targeting system. Physicochemical characterization provided indirect evidence consistent with surface functionalization through zeta potential and Nanodrop measurements, whereas FTIR confirmed retention of the core POSS, carbonyl, and RITC-associated chemistry without demonstrating aptamer conjugation. Biological evaluation in a PC-3 prostate cancer and THP-1-derived macrophage co-culture model demonstrated concentration-dependent changes in apoptotic distribution patterns, which should be considered preliminary interaction-associated response profiles rather than direct evidence of cytotoxicity, receptor-specific binding, or immune checkpoint modulation. The most pronounced responses were observed within a defined low-dose treatment window, while higher concentrations did not further enhance the effect.

These findings document cell-associated response patterns within the tumour–macrophage co-culture system, but the present controls do not establish that nanoscale architecture, ligand presentation, or sequence-dependent CD47 activity caused the observed effects. The observed effects are consistent with interaction-associated cellular redistribution but do not constitute direct evidence of receptor-specific targeting or immune checkpoint modulation. Instead, the results should be interpreted as indirect functional indications of nanoparticle–cell interaction dynamics under the tested conditions.

Beyond CD47-oriented systems, the modular architecture, green fabrication strategy, and optically traceable character of the RITC@MMES-POSS platform support its potential utility as a biomimetic and biohybrid materials platform for biomedical interface studies, including bioimaging-oriented applications and ligand-guided cellular interaction models. The modularity of its surface chemistry and ligand-conjugation strategy supports adaptation to alternative biological targets and experimental systems, although improved control of colloidal size and dispersion stability will be required in future studies.

Future studies will focus on direct validation of targeting specificity using surface-sensitive techniques such as XPS, quantitative assessment of macrophage-related functions, and mechanistic elucidation using advanced in vitro and in vivo models. Collectively, this work provides a scalable, modular, and optically traceable biohybrid nanoplatform for preliminary investigation of tumour–immune interaction patterns, rather than establishing a definitive therapeutic mechanism.

## Figures and Tables

**Table 1:** Polymerization composition.

Components	RITC@MMES-POSS
Water Phase	
H_2_O (mL)	10
SDS (g)	0.025
Organic Phase	
M-POSS (g)	0.025
MMES (g)	0.025
Darocur 1173 (mL)	0.020
Ethanol (mL)	1.000

**Table 2:** Experimental (exp.) and control groups.

Groups	Content
NC1	PC-3 + Medium
NC2	THP-1 + Medium
NC3	PC-3 + THP-1 + Medium
NC4	PC-3 + THP-1 + RITC@MMES-POSS
Exp	PC-3 + THP-1 + Apt-RITC@MMES-POSS
PC	PC-3 + THP-1 + anti-CD47

**Table 3:** Annexin V–FITC/PI population distribution in the PC-3/THP-1 co-culture model under different treatment conditions. Values for the 0.0625–128 µg/mL concentrations and the positive control represent mean ± SD from three technical replicates; the untreated co-culture value is a single measurement and therefore has no associated SD.

**Amount (µg/mL)**	**Q1 (Necrotic)**	**Q2 (Late Apoptotic)**	**Q3 (Early Apoptotic)**	**Q4 (Viable)**
0.0 (Untreated co-culture)	0.53	48.35	11.75	39.35
0.0625	5.24 ± 0.76	29.22 ± 4.21	16.39 ± 0.92	49.15 ± 4.92
0.125	9.05 ± 1.79	42.20 ± 3.47	13.95 ± 0.58	34.80 ± 3.33
0.25	2.56 ± 1.62	18.42 ± 4.91	4.96 ± 1.42	74.07 ± 7.70
0.50	1.93 ± 0.77	41.21 ± 6.02	21.40 ± 2.45	35.46 ± 8.29
1.0	2.72 ± 0.94	26.93 ± 4.28	27.14 ± 2.51	43.22 ± 5.56
2.0	0.53 ± 0.14	38.67 ± 2.03	36.61 ± 12.69	24.19 ± 10.91
4.0	0.57 ± 0.73	18.36 ± 3.00	14.49 ± 2.63	66.58 ± 3.42
8.0	4.40 ± 0.96	26.31 ± 0.83	16.51 ± 1.93	52.79 ± 0.67
16.0	2.39 ± 0.44	24.06 ± 2.79	20.39 ± 4.05	53.16 ± 6.23
32.0	1.31 ± 1.22	27.67 ± 2.12	18.69 ± 3.95	52.32 ± 4.32
64.0	2.63 ± 1.04	20.19 ± 3.82	15.39 ± 1.57	61.79 ± 5.85
128.0	1.45 ± 0.41	19.41 ± 4.26	11.17 ± 1.25	67.97 ± 4.57
**Positive control**	6.30 ± 2.74	28.95 ± 7.90	21.33 ± 2.06	43.42 ± 7.19

## Data Availability

The original contributions presented in this study are included in the article/Supplementary Material. Further inquiries can be directed to the corresponding authors.

## Supplementary Materials

The following supporting information can be downloaded at https://www.mdpi.com/article/10.3390/biomimetics11080588/s1, Figure S1. Comparison of apoptotic profiles of PC-3 and THP-1 cells under monoculture and co-culture conditions; Figure S2. apoptotic profiles of negative control groups in the PC-3/THP-1 co-culture system; Figure S3. Cytotoxicity assessment of aptamer-functionalized nanoparticle treatments and control treatments in the PC-3/THP-1 co-culture system; Figure S4. RITC-channel fluorescence histogram overlays and MFI summary across all tested concentrations and control groups; Table S1. Energy-dispersive X-ray spectroscopy (EDX) results; Table S2. DLS characterization and summary parameters for RITC@MMES-POSS nanoparticles dispersed in water; Table S3. Omnibus one-way ANOVA and exploratory pairwise Welch’s *t*-test results for the Annexin V-FITC/PI population distribution.

## Abbreviations

The following abbreviations are used in this manuscript:

CD47	Cluster of Differentiation 47
SIRPα	Signal-regulatory protein alpha
POSS	Polyhedral oligomeric silsesquioxane
MMES	Mono-2-(methacryloyloxy)ethyl succinate
RITC	Rhodamine-B-isothiocyanate
SEM	Scanning electron microscope
EDX	Energy-dispersive X-ray spectroscopy
FTIR	Fourier transform infrared
FITC	Fluorescein isothiocyanate
PI	Propidium iodide
SDS	Sodium Dodecyl Sulfate
NHS	N-hydroxysulfosuccinimide
EDC	1-ethyl-3-(3-dimethylaminopropyl)carbodiimide
EDAC	1-ethyl-3-(3-dimethylaminopropyl) carbodiimide hydrochloride
PMA	Phorbol 12-myristate 13-acetate
Si	Silicon
O	Oxygen
C	Carbon
N	Nitrogen
P	Phosphorus
