# Supplementary material for: CD47 Aptamer-Decorated Fluorescent POSS Hybrid Nanoparticles as a Biohybrid Interface for Probing Prostate Cancer–Macrophage Interactions
Source: Biomimetics (Basel). 2026 Aug 18;11(8):588. doi: 10.3390/biomimetics11080588 (PMC13511493; doi:10.3390/biomimetics11080588)
Supplement: Supplementary file 1 [file biomimetics-11-00588-s001.zip › biomimetics-4469037-supplementary.pdf]

CD47 Aptamer-Decorated Fluorescent POSS Hybrid Nanoparticles as a Biohybrid Interface for Probing Prostate Cancer–Macrophage Interactions

Sumeyye Altunok [1,2,†], Gunes Kibar [1,3,†], Fatma Aylaz [1], Dide Su Demirel [1] and Veli Cengiz Ozalp [4,*]

[1] Medical School, Faculty of Medicine, Atılım University, 06830 Ankara, Türkiye; altunok.1@osu.edu (S.A.); gkibar@atu.edu.tr (G.K.); fatma.aylaz@atilim.edu.tr (F.A.); demirel.didesu@student.atilim.edu.tr (D.S.D.)

[2] Department of Neurology, The Ohio State University Wexner Medical Center, Columbus, OH 43210, USA

[3] Micro Nano Particles (MNP) Research Group, Department of Materials Science and Engineering, Adana Alparslan Türkeş Science and Technology University, 01250 Adana, Türkiye

[4] Department of Biomedical Engineering, Faculty of Engineering and Natural Sciences, İstanbul Atlas University, 34408 İstanbul, Türkiye

* Correspondence: cengiz.ozalp@atlas.edu.tr

† These authors contributed equally to this work.

Supplementary Tables and Figures

Supplementary Table S1. Energy-dispersive X-ray spectroscopy (EDX) results

Nanoparticles Name	Element (%)					
	C		O		Si	
	Wt.	At.	Wt	At.	Wt.	At.
**RITC@MMES-POSS**	51.25	65.85	17.74	17.11	31.01	17.04
**Modified RITC@MMES-POSS**	54.14	68.02	18.07	17.04	27.80	14.94
**Apt-RITC@MMES-POSS**	59.31	70.18	24.16	21.46	16.52	8.36

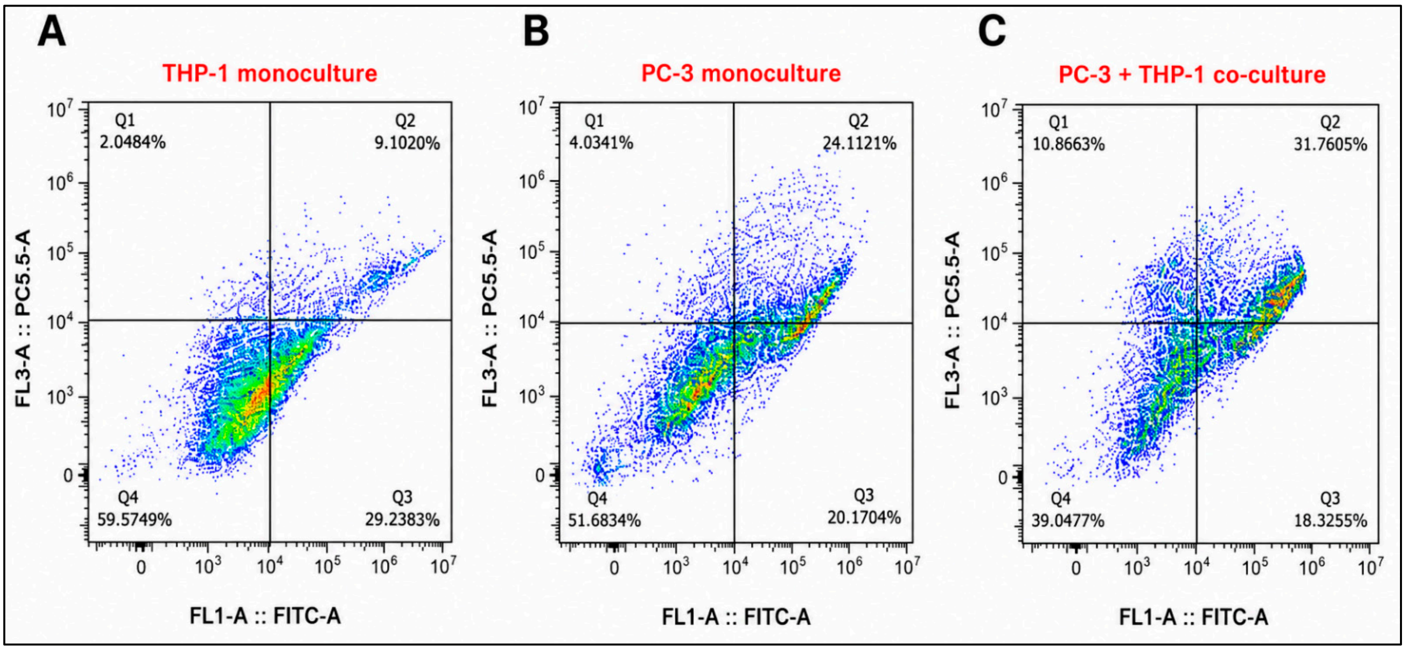

Supplementary Figure S1. Comparison of apoptotic profiles of PC-3 and THP-1 cells under monoculture and co-culture conditions. (A) NC1 group (PC-3 + medium). Annexin V–FITC/PI flow cytometric analysis of PC-3 cells cultured under monoculture conditions. The majority of the cell population is localized in the Annexin V^{-}/PI^{-} (viable) quadrant, indicating low basal levels of apoptosis. **(B)** NC2 group (THP-1 + medium). Apoptotic profile of THP-1 cells under monoculture conditions. Compared with NC1, a distinct distribution pattern is observed, reflecting cell type–specific basal apoptotic characteristics. **(C)** NC3 group (PC-3 + THP-1 + medium). Apoptotic profile of PC-3 and THP-1 cells under co-culture conditions. In comparison with monoculture controls (NC1 and NC2), a pronounced shift in cell population distribution is observed, indicating altered apoptotic dynamics and confirming effective cell–cell interactions between PC-3 and THP-1 cells.

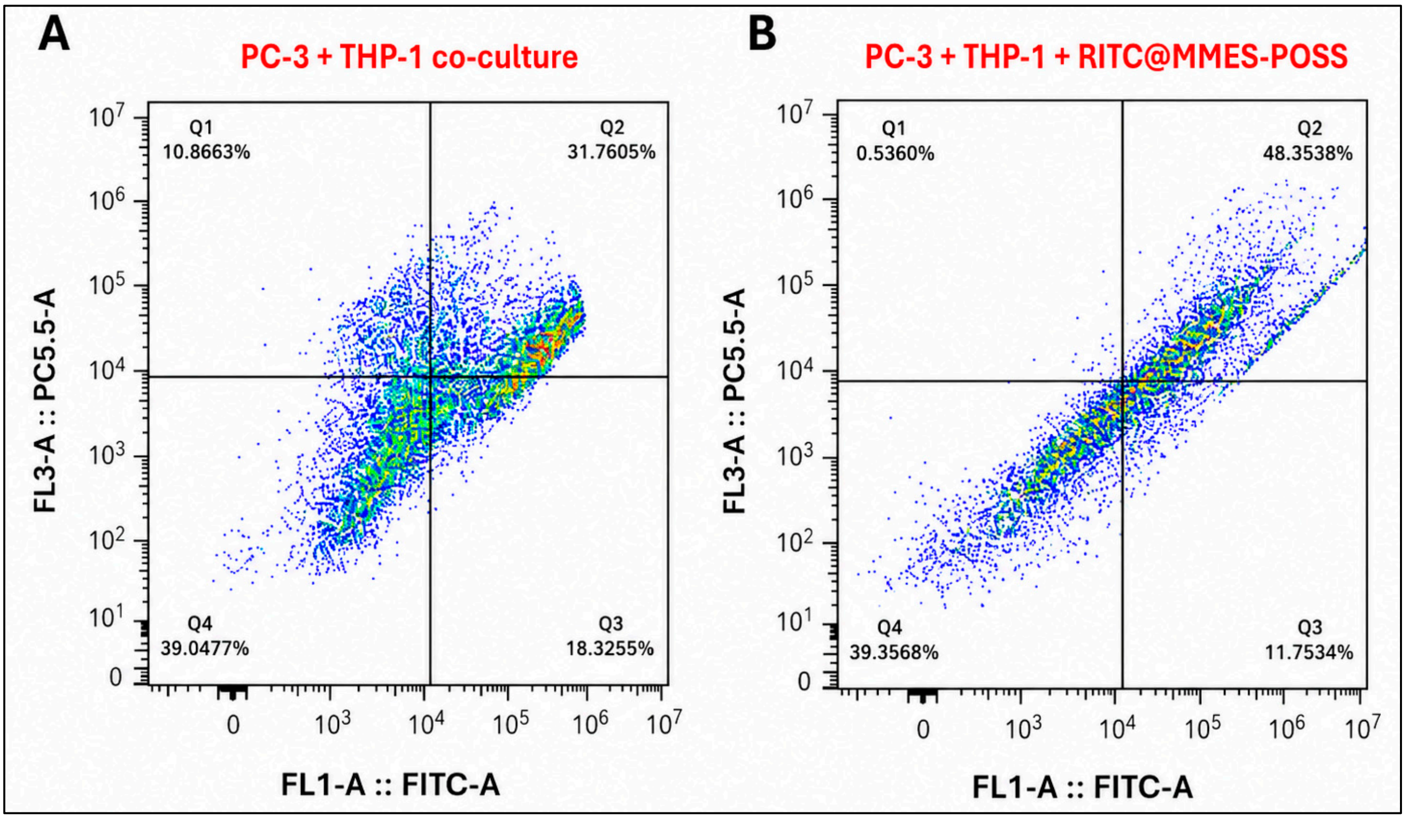

Supplementary Figure S2. **Apoptotic profiles of negative control groups in the PC-3/THP-1 co-culture system.** Representative Annexin V–FITC/PI flow cytometric dot plots are shown. **(A)** NC3 group (PC-3 + THP-1 + medium). Apoptotic profile of cells under untreated co-culture conditions. **(B)** NC4 group (PC-3 + THP-1 + RITC@MMES-POSS). Apoptotic profile of cells in the co-culture system following treatment with aptamer-free RITC@MMES-POSS nanoparticles.

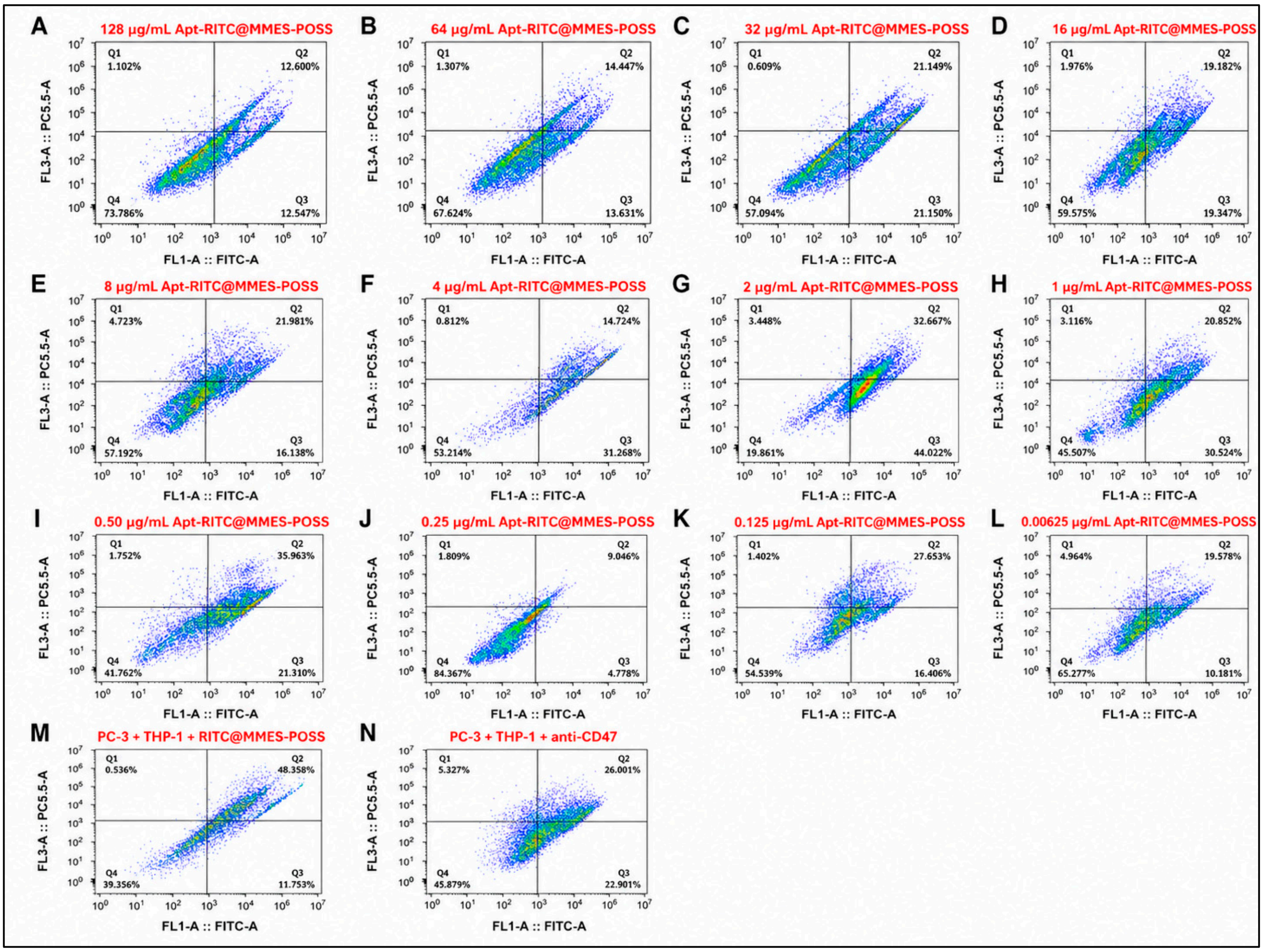

Supplementary Figure S3. Cytotoxicity assessment of aptamer-free and control treatments in the PC-3 + THP-1 co-culture system. Representative Annexin V–FITC/PI flow cytometric dot plots showing apoptotic profiles of PC-3 cells co-cultured with PMA-differentiated THP-1 macrophages under different treatment conditions. **(A-L)** PC-3 + THP-1 + APT-RITC@MMES-POSS nanoparticles at increasing concentrations of 128, 64, 32,16, 8, 4,2, 1, 0.5, 0.25, 0.125, and 0.0625 μg/mL, respectively. **(M)** Negative nanoparticle control: PC-3 + THP-1 + RITC@MMES-POSS nanoparticles. **(N)** Positive control: PC-3 + THP-1 + CD47 antibody, demonstrating enhanced apoptotic responses.

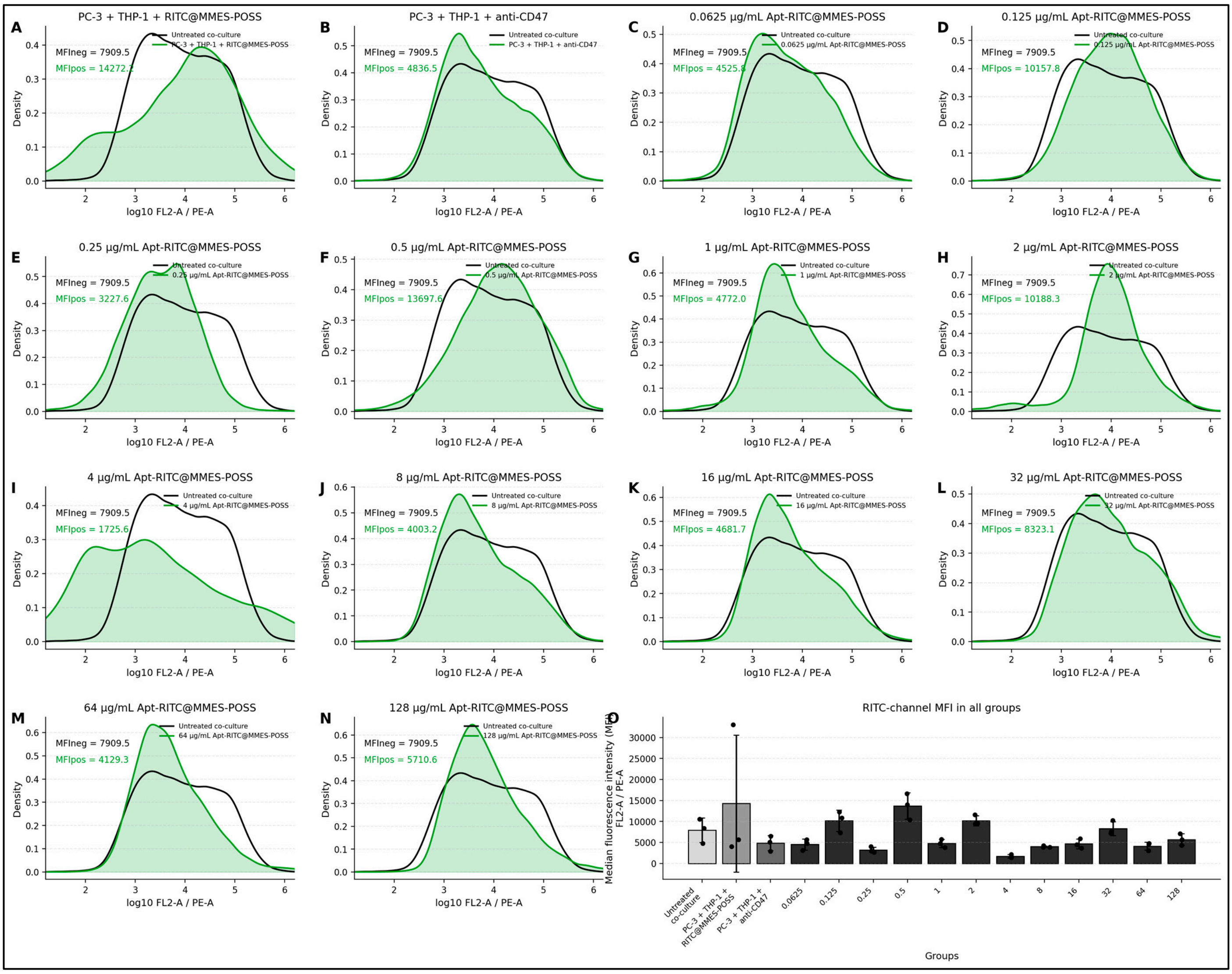

Supplementary Figure S4. Overlay RITC-channel fluorescence histograms and summary MFI analysis for all tested concentrations and control groups in PC-3/THP-1 co-cultures. Representative RITC-channel histogram overlays are shown for the untreated co-culture, RITC@MMES-POSS nanoparticle control, anti-CD47-treated positive control, and Apt-RITC@MMES-POSS-treated groups across the full concentration range (0.0625–128 µg/mL). Untreated co-culture controls are shown in black, and treated groups are shown in green. The summary bar graph presents RITC-channel median fluorescence intensity (MFI) values calculated from the FL2-A/PE-A channel and expressed as mean ± SD from technical replicates. These data provide a comparative overview of fluorescence-associated cellular changes across the full treatment series and were interpreted as supportive evidence of nanoparticle-associated fluorescence rather than definitive proof of receptor-specific CD47 binding.

Supplementary Table S2. Dynamic light scattering (DLS) characterization of RITC@MMES-POSS nanoparticles dispersed in water (n = 3 independent measurements).

Replicate	Size(d.nm)	St. Dev (d.nm)	PDI
1 (CD 1)	44.06	4.57	1.000
2 (CD 2)	177	22.54	1.000
3 (CD 3)	244.1	30.38	0.891

Measurements were performed on a Malvern Zetasizer (Ver. 7.12, Serial No. MAL1062728) at 25 °C using water as the dispersant. For all three replicates, the automated result-quality assessment flagged the cumulant fit ("Refer to quality report"), and PdI values approached or equaled 1.0, indicating a broad, polydisperse and/or partially aggregated population in the hydrated state. These DLS values are substantially larger than the dry-state diameters measured by SEM (~70 nm), consistent with hydration, and partial aggregation.

Supplementary Table S3. Statistical analysis of the Annexin V–FITC/PI population distribution (Table 3) across the 0.0625–128 μg/mL concentration series. (A) One-way ANOVA testing the overall effect of nanoparticle concentration on each Annexin V/PI quadrant population (Q1–Q4) across all 12 tested concentrations (df = 11, 24). (B) Selected pairwise Welch's t-test comparisons discussed in the main text.

(A) One-way ANOVA summary

Quadrant	F(11,24)	p-value
Q1 (Necrotic)	16.0	< 0.0001
Q2 (Late apoptotic)	15.6	< 0.0001
Q3 (Early apoptotic)	10.6	< 0.0001
Q4 (Viable)	18.7	< 0.0001

(B) Selected pairwise Welch's t-test comparisons

Comparison	Quadrant	p-value
0.25 μg/mL vs. 0.125 μg/mL	Q4 (Viable)	0.0056
0.25 μg/mL vs. 0.5 μg/mL	Q4 (Viable)	0.0042
0.125 μg/mL vs. 0.5 μg/mL	Q4 (Viable)	0.91
2 μg/mL vs. 1 μg/mL	Q3 (Early apoptotic)	0.32
2 μg/mL vs. 4 μg/mL	Q3 (Early apoptotic)	0.075
1 μg/mL vs. anti-CD47 positive control	Q3 (Early apoptotic)	0.038
1 μg/mL vs. aptamer-free NP control	Q3 (Early apoptotic)	0.059
